# Scenario-Based Simulation Modeling for Performance and Efficiency Improvement in an Ultrasonography Department

**DOI:** 10.3390/healthcare14060709

**Published:** 2026-03-10

**Authors:** İlkay Saraçoğlu

**Affiliations:** Department of Industrial Engineering, Haliç University, Istanbul 34060, Turkey; ilkaysaracoglu@halic.edu.tr

**Keywords:** health services, ultrasound, IoT, simulation optimization, healthcare performance indicators

## Abstract

**Background/Objectives**: Hospitals prioritize effective resource allocation and patient satisfaction as key performance indicators. Improving the performance of the ultrasonography department remains a major challenge for hospital management due to the inherently unplanned and stochastic nature of its operations. Arrival patterns vary throughout the day, and examination durations differ depending on patients’ clinical pathways and examination types. This study focuses on the ultrasonography department of a private healthcare facility located in one of the most densely populated regions of Istanbul. The primary objective of this study was to improve departmental performance in terms of average waiting time, total time spent in the system, and resource utilization. **Methods**: To address the variability in patient arrivals and service times across different ultrasonography procedures, a simulation-based optimization approach was employed. Current system performance was evaluated, and multiple alternative operational scenarios were developed and simulated. In addition, the potential impact of Internet of Things applications on the performance of the ultrasonography department was investigated by incorporating alternative system configurations into the simulation model. **Results**: The simulation results enabled a comparative evaluation of alternative scenarios based on key performance indicators. The findings demonstrate that optimized system configurations can significantly reduce patient waiting times and total system time while improving resource utilization. The inclusion of Internet of Things applications further contributed to performance improvements in the selected scenarios. **Conclusions**: The proposed simulation-based approach provides a systematic decision-support framework for evaluating alternative operational scenarios in ultrasonography departments. By optimizing resource allocation and leveraging Internet of Things applications, hospital managers can improve operational efficiency and patient satisfaction. The results highlight the value of data-driven decision-making in managing complex and stochastic healthcare systems.

## 1. Introduction

The management of healthcare services in hospitals is becoming more important. Hospitals strive to cut costs and boost financial assets while simultaneously improving patient satisfaction. Patient waiting times are a crucial performance indicator for hospitals in terms of patient satisfaction [1,2]. Patient waiting times (measured as average wait time (AWT)), the number of patients waiting in the hospital (WIP), patient time spent in the system (PST), and resource utilization are all valuable indicators for hospital management [3,4]. The use of appointment systems in recent years has helped to reduce patient wait times. In departments without an appointment system, improving performance criteria can be challenging due to unpredictable patient arrivals and the uncertainty of required procedures. The healthcare sector is increasingly being recognized as a complex industrial system in which patient flows, stochastic demand and limited resources must be managed simultaneously; consequently, discrete event simulation (DES) has become a widely used tool for process improvement and decision support in this setting [5]. A recent systematic review of DES applications in healthcare reported that 25.8% of case studies were conducted in emergency departments, followed by generic hospital models and oncology, operating rooms, cardiology, and neurology, with a smaller but growing number of studies focusing on diagnostic imaging, including ultrasound departments [6]. In the same review, it was found that 31.8% of DES applications primarily targeted a reduction in waiting times, 24% a reduction in service costs, and 18% an increase in service capacity, with many case studies achieving multiple improvements simultaneously. DES is described as a powerful decision-support technique that allows stakeholders to conduct “what-if” experiments on models representing real systems in order to forecast the impact of alternative strategies or policies without intervening in real patient care. In this study, the primary motivation for adopting DES for the ultrasound department is to exploit these documented benefits. Stochastic processes such as patient arrivals and examination and reporting times are represented by appropriate statistical distributions, and then “what-if” analysis is used to safely test alternative capacity and workflow configurations in a virtual environment, thereby enabling evidence-based comparison of scenarios that aim to reduce waiting times, lower service costs and improve capacity.

Simultaneously optimizing all performance indicators is inherently challenging. For instance, minimizing patient waiting time may require increasing the number of available resources, but this may lead to higher operational costs and lower resource utilization rates. Therefore, improvements in one indicator may lead to a deterioration in another. To this end, different operational scenarios have been developed, and a methodology has been proposed to enable hospital managers to make decisions by evaluating these scenarios using TOPSIS, a multi-criteria decision-making method.

Ultrasound plays a crucial role in the treatment and diagnosis of an extensive array of disorders. However, due to their high cost and limited availability, it is crucial to use them wisely. Implementing this solution may effectively manage the movement of patients, minimize wait durations, and maximize the availability of medical practitioners for treatment. Historical data shows that approximately 99% of ultrasound referrals originate from outpatient clinics. Although patients attend the hospital with scheduled outpatient appointments, ultrasound referrals are typically decided during the consultation and are not arranged in advance. This practice introduces significant demand uncertainty and arrival variability in the ultrasound department, as both the timing and the volume of incoming patients cannot be precisely predicted from an operational standpoint. The objective of this research is to enhance effectiveness by examining data from the ultrasound department of a private healthcare institution in the densely populated region of Istanbul.

The objective of this research is to enhance effectiveness by examining data from the ultrasound department of a private healthcare institution in the densely populated region of Istanbul.

Sound waves are used extensively in many applications within the domains of science, medicine, and technology. Ultrasonography is a prevalent medical imaging technology used to diagnose disorders in anatomical regions that are difficult to reach. This technique offers diagnostic and therapeutic possibilities for checking organs in the abdominal region, performing breast examinations, and monitoring the development of pregnancy. The key areas of ultrasonography usage include the following:

- Assessing the health status of the fetus during pregnancy;
- Identifying potential irregularities in heart function;
- Screening for different types of infections;
- Identifying gallstones and related conditions;
- Examining breast and soft tissue for tumors;
- Evaluating conditions related to muscle disorders;
- Investigating diseases affecting the prostate and genital area;
- Providing imaging guidance for procedures such as cyst aspirations and biopsies;
- Assessing diseases related to the thyroid gland.

In this study, a simulation model is developed that evaluates the current system. In addition, an alternative system design is proposed and different operational scenarios are examined to assess the impact of IoT device implementation on key performance indicators such as patient waiting time, ongoing workload, time spent in the ultrasound department, and resource utilization.

The remainder of this paper is organized as follows: Section 2 presents a comprehensive summary of the relevant literature. Section 3 provides a detailed explanation of the techniques used in this study, including the process of collecting data, creating simulation models, and presenting the findings. Finally, Section 4 provides definitive findings and suggestions for future research.

## 2. Literature Review

The primary focus of this study is a simulation-based evaluation of operational improvement alternatives in an ultrasound department, and its primary objective is to develop a scenario-based framework that supports managerial decision-making under demand uncertainty. In addition to these operational scenarios, a limited number of forward-looking configurations are included to demonstrate how emerging digital health technologies might impact system performance. These technology-focused extensions are included for exploratory purposes and to highlight potential future research directions, rather than to position digital solutions as the central contribution of the study.

The initial focus of the literature review was the use of simulation methods in hospitals. Due to the variability in patient arrival and service times in healthcare facilities, most researchers have undertaken simulation-based studies [7]. These studies primarily concentrate on the emergency department. For instance, Wang et al. [8] examined a hospital’s emergency department using simulation models to optimize patient wait times and enhance physician efficiency. Similarly, Ruohonen et al. [9] improved emergency department processes by introducing a new triage team through simulation. Additionally, Gharahighehi et al. [10] and Haghighinejad et al. [11] attempted to shorten patient wait times by modeling different scenarios for the emergency department. Corsini et al. [12] developed an agent-based simulation model designed for oncology departments working with external pharmacies to optimize patient flow and minimize wait times. Aboueljinane and Frichi [13] proposed a simulation optimization methodology to improve resource planning choices and ambulance relocation in emergency departments. They also explored the advantages of coordinating various stakeholders for joint decision-making. Liu et al. [14] considered uncertainty and proposed an innovative approach featuring the use of simulation techniques to address resource scheduling challenges in emergency departments. Five strategies, including “FIFO + Centralised” and “Random + Centralised,” were implemented and compared in terms of patient stay duration efficiency. Vázquez-Serrano et al. [15] developed a hybrid model based on optimization and discrete-event simulation, specifically considering how patient priority, preparation time, and resource availability could be optimized to reduce waiting time.

There have been numerous studies on organizing patient appointment systems in the healthcare field, often in conjunction with emergency service simulation, to enhance patient satisfaction levels. Moreno and Blanco [16] considered two main objectives: granting early admittance to high-priority patients and reducing hospitalization durations. Their research utilized a fuzzy programming technique to tackle randomness in clinical service availability. Jlassi et al. [17] presented a mathematical model using integer programming to reduce the overall waiting time for patients, considering limitations related to procedures and personnel availability. Genetic Algorithms (GAs) have been developed as solution methods for handling large-scale problems. On the other hand, one of the key issues in healthcare services is patient prioritization. Frichi et al. [18] proposed a dynamic technique to prioritize elective surgery patients. Their method entails prioritizing patients according to their evolving health condition, wait duration, and the likelihood of patients choosing to withdraw from the waiting list. It uses the Analytic Hierarchy Process (AHP) to provide weights for ranking criteria. The weights are implemented into a discrete-event simulation model, which systematically evaluates patients based on their changing health status and waiting times and subsequently assigns them a ranking.

This article focuses on the ultrasound department, identified as a challenging area based on interviews with hospital management. A literature review was conducted, and though studies summarizing patient waiting times in the radiology department were found, they revealed a lack of studies addressing waiting time reduction specifically in ultrasound departments. That said, Shakoor [19] developed a simulation model to assess ultrasound department performance under different appointment scheduling policies, and Chen et al. [20] created a simulation model to assess how different appointment scheduling policies impact the performance of the ultrasound department. The emphasis remains on appointment scheduling optimization under uncertainty. In another study, Chen et al. [21] sought to identify the optimal inter-arrival time between patients in order to maximize patient throughput and minimize waiting time. In a recent study on the ultrasound department, machine learning methods were applied to accurately predict patient arrivals. Based on these forecasts, the required number of physicians on an hourly basis was determined, and physicians’ working schedules were subsequently arranged using an optimization model [22]. The emergence of Industry 4.0 has brought technological advancements to healthcare, as seen in Kadarla et al.’s [23] exploration of IoT technology in healthcare settings. They proposed a heuristic approach to optimize resource utilization in cloud environments for IoT healthcare applications. Kumar et al. [24] discussed Healthcare 4.0, incorporating IoT, AI, and cloud computing, emphasizing the validation of data access processes through simulation and optimization methods. To demonstrate the impact of wearable breast ultrasound devices developed by Du et al. [25] and Luo et al. [26] on patient waiting times, this study compared current practices with scenarios where patients could self-administer ultrasounds. Saraçoǧlu and Elgörmüş [27] considered the use of IoT applications to reduce patient waiting times in the ultrasound department; however, their evaluation was limited to the current system configuration.

Generally, the existing literature focuses primarily on emergency departments, where patient flow optimization and waiting time reduction have been extensively examined. In contrast, despite their operational complexity and increasing demand, there are relatively few studies specifically addressing ultrasound departments. Furthermore, studies in this context primarily focus on appointment scheduling improvements, particularly time slot adjustments, focusing on the situation in which patients arrive at the ultrasound department with an appointment. This study differs from previous research by evaluating how alternative operational designs in the ultrasound department affect overall system performance. It also assesses the performance of ultrasound departments when patients arrive without an appointment. Moreover, it includes an assessment of the impact of new technological developments that have not yet been systematically examined in the literature but are likely to shape healthcare operations in the coming years. In other words, it differs from other studies by drawing attention to the impact of new technological developments on the system that have not been addressed in previous studies but should be considered in the future.

## 3. Methodology

### 3.1. Data Analysis and Evaluation of Current System

**Figure 1:**
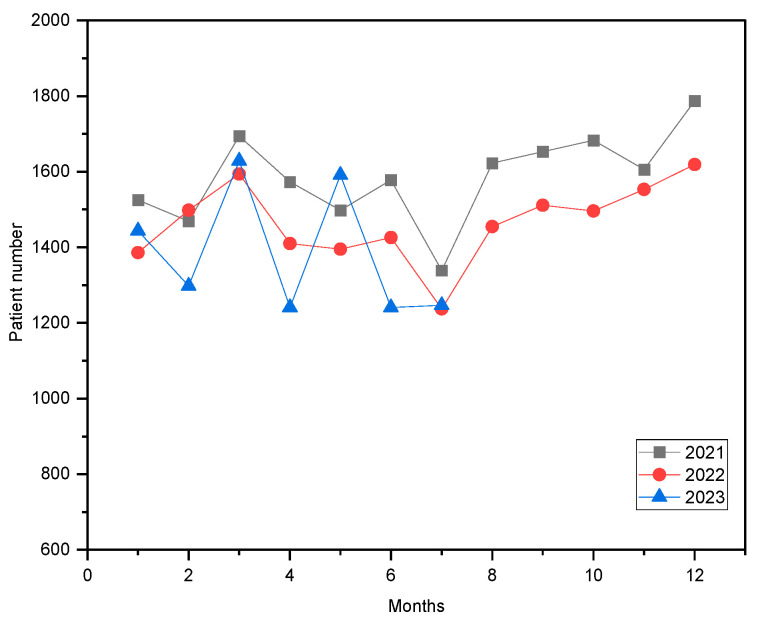
Monthly total patient numbers in the US department.

This study examined patients’ historical data from the hospital’s ultrasound service from January 2021 to August 2023. People make arrangements to go to the hospital’s outpatient offices. If a patient needs treatment in the US, the doctors fill out a form to send them to the US department. The patient goes to the US department for appropriate treatment after a request is sent. Once a report with the results is available, the patient returns to their doctor to review the findings. Firstly, changes in patient arrival numbers by year were examined to decide whether there is a trend or not. The monthly total number of patients is presented in Figure 1 (by year). Additionally, the years 2021 and 2022 may still be considered to have been under the influence of the COVID-19 pandemic.

COVID-19 protection measures in Turkey were officially lifted in March 2022, and all international flight restrictions were lifted in June 2022. To address this issue in more detail, we conducted statistical analyses to examine whether there were significant differences in patient arrival patterns between years within the study period. The results showed no statistically significant structural differences in arrival distributions between years. Additionally, our dataset includes patient arrivals from the complete lifting of restrictions until August 2023.

The descriptive statistical data shown in Table 1 summarizes the yearly distribution of patients in the US department. For 2020, the average number of patients who visited the US department is 40.84, and the standard deviation is 20.40. For 2021 and 2022, the average daily number of patients is 44.44 and 41.41, respectively, and the standard deviation is 18.59 and 16.62, respectively. It can be seen that, on average, 39.28 people visited the department every day in the first seven months of 2023, with 16.16 being the standard deviation. Statistical analysis was performed to analyze the difference between the years according to the number of patients coming to the US department daily. The *p*-value was found to be 0.155, indicating that there is no significant difference in the number of patients arriving in the ultrasound department across the years (95% confidence level).

**Figure 2:**
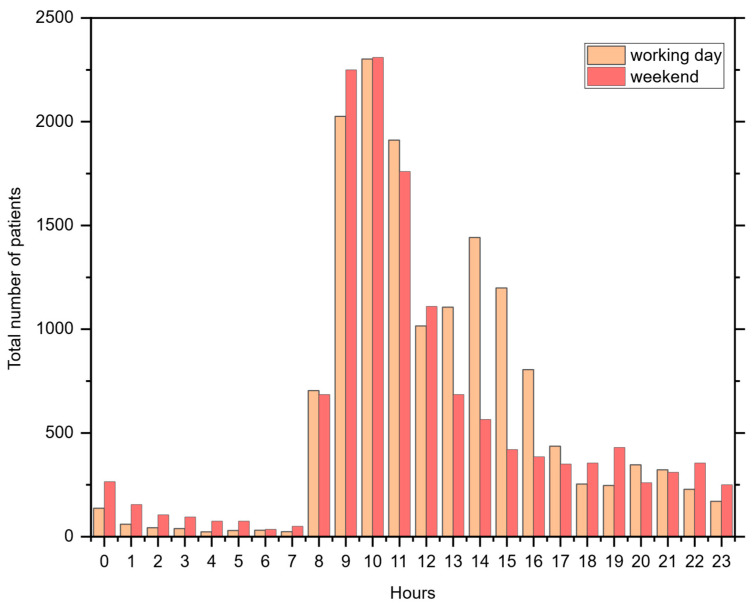
Hourly data regarding patients served in the US department.

The data revealed no significant change in the daily number of patients entering the department throughout the studied years. The variation between different days of the week was also examined. Figure 2 illustrates hourly data regarding patient visits to the US department on weekdays and weekends.

Table 2 compares the number of patients that visit the US department on weekdays and weekends. While 58.41 patients visited the department on weekdays, 25.09 patients did so on weekends. These analyses were performed to design and decide the simulation period in simulation modeling.

**Figure 3:**
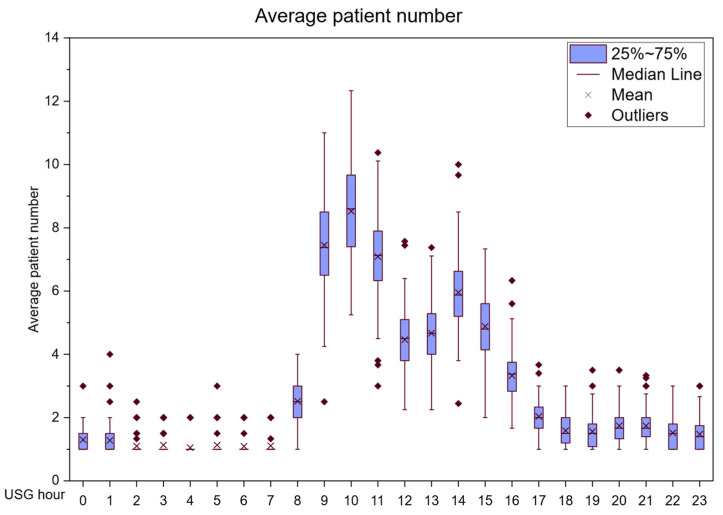
Hourly arrival rates of patients.

An investigation into whether there were differences between certain weekdays, from Monday to Friday, was also conducted. An ANOVA analysis revealed that there was no discernible difference between years, months, and weekdays. A difference was found between the number of patients arriving on weekdays and Saturdays and Sundays. The differences between hours in the day were not investigated. The number of patients entering the US department in each time zone is shown in Figure 3.

Figure 3 shows that the peak arrival of patients occurs between 10:00 and 11:00. After 18:00, the number of patients significantly declines. Table 3 shows a statistically significant difference in hourly patient arrivals at the ultrasonography department.

### 3.2. Decision of the Distribution of Variables

Distribution goodness-of-fit tests were carried out to facilitate the use of patient interarrival times and processing times, which are variables whose variability is statistically proven, for the simulation model [26]. According to Chi-Square tests, the corresponding *p*-value was 0.578 for arrival rates between 10:00 and 11:00, the busiest time. Distribution goodness-of-fit was applied to the interarrival time of the patients for all time periods, and the distributions obtained are summarized for each time period in Table 4. For experimental factors, a *p*-value below 0.05 signifies a statistically significant effect on the expected mean flow time at a 95% confidence level. We selected the most frequently performed operations in the US department by analyzing 930 days of historical data. Since the durations of these operations varied, we applied a goodness-of-fit test to the distribution of each process. The Arena Input Analyzer was employed to assess the time taken to perform each type of ultrasound (US) procedure listed in Table 5, as derived from this analysis.

Patients are referred to the ultrasound (US) department from several sources, including the emergency department, critical care unit, and inpatient and outpatient clinics. Historical data shows that approximately 99% of patients who come to the US department are referred from outpatient clinics. Although these patients typically attend the hospital with scheduled outpatient clinic appointments, ultrasound referrals are generated during the clinical visit and are not pre-scheduled; therefore, the exact timing and volume of arrivals to the US department remain ambiguous from an operational perspective.

According to hospital management and US physicians, the department employs four doctors. Three doctors work from 9:00 A.M. to 6:00 P.M. on weekdays, while one doctor works from 9:00 A.M. to 2:00 P.M. Therefore, four physicians are available between 9:00 A.M. and 2:00 P.M., after which the number of available physicians decreases to three for the remainder of the working day. This mid-shift reduction in physician capacity was incorporated into the simulation model using the resource scheduling feature of the simulation software. In cases where an emergency patient arrives, and all physicians are occupied, ultrasound procedures are conducted in another area of the hospital containing an ultrasound device.

### 3.3. Simulation Model and Results

**Figure 4:**
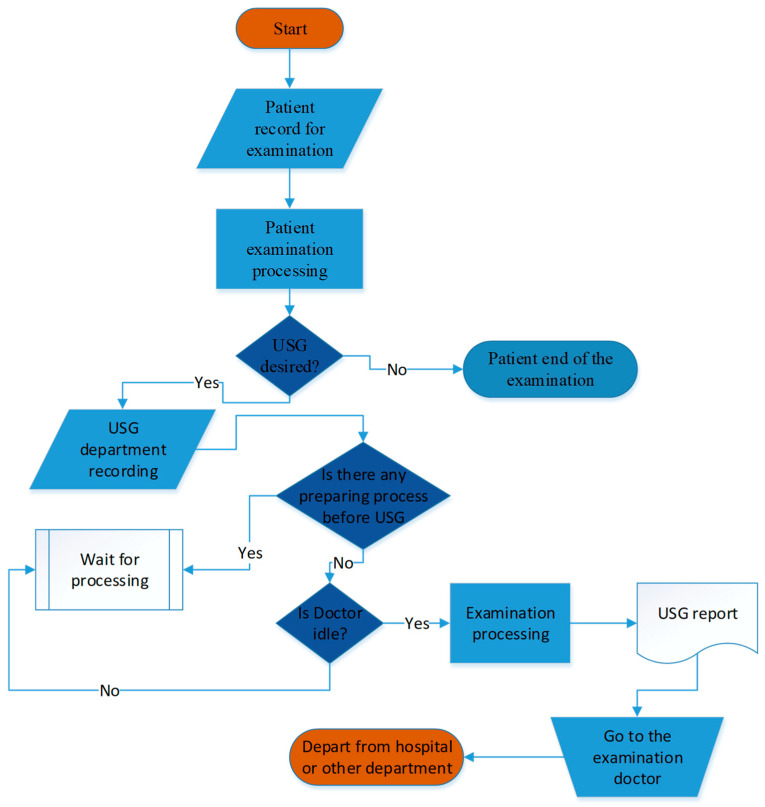
Flow chart for patient arrival system.

After examination, doctors can refer the patient to the US department for treatment. Subsequently, the patient proceeds to the US department’s registration desk to make payment. The secretary then reviews the patient’s procedure and makes referrals based on doctor availability and preliminary preparation requirements. The planning of an ultrasound procedure starts with assessing which doctors are available. The department features four examination rooms, each equipped with a doctor, an assistant, and an ultrasound machine. The assistant prepares the patient for the procedure, captures vocal notes on the computer, and creates the report. Once the ultrasound is finished, the patient goes back to the examination room to review the report. For procedures involving abdominal ultrasound and the urinary system, the patient is expected to have a full bladder. This process flow is shown in Figure 4.

The simulation study was carried out in Rockwell’s Arena Simulation software, version 16.0. The simulation was analyzed using an Intel(R) Core (TM) i7-10510U CPU@1.80GHz 2.30GHz computer. The study used discrete-event modeling, with the hospital operating for 9 h (540 min) each day. The goal of the study is to improve the performance of the US department. The performance evaluation criteria for hospital departments included average patient waiting times, number of patients served, resource utilization rates, and patient time spent in the system. A simulation model has been established to measure these criteria under current conditions. To assess the validity of the simulation model, the simulation outputs were compared with the observed performance of the real system. According to actual data obtained from the ultrasound department, the average patient waiting time in the real system is 45.31 min. Whether the created simulation model is suitable for the real system should be tested during the validation phase [28]. To assess the validity of the simulation model, the simulation outputs were compared with the observed performance of the real system. According to actual data obtained from the ultrasound department, the average patient waiting time in the real system is 45.31 min. When the simulation was run with 30 replications, the estimated average waiting time was 37.73 min, with a standard deviation of 26.44 min and a standard error of 4.83 min, resulting in a 95% confidence interval of (27.85, 47.60) minutes. To validate the model, it was compared to the real system using a *t*-test at a 95% confidence level. The hypotheses for the validation test were as follows:


$$H_{0}=E\left(avg.waiting\;time\right)\;=45.31\;minutes$$

versus

$$H_{1}=E\left(avg.waiting\;time\right)\;\neq 45.31\;minutes.$$

The significance level (α) and the number of replications (n) were set at 0.05 and 10, respectively. The *t*-value was determined to be $t_{0.025,29}=2.045\;$ based in the *t*-table. The test statistics were calculated as follows:

$$t_{0}=\frac{avg.waiting\;time-\mu_{0}}{S/\sqrt{n}}=\frac{40.12-45.31}{26.44/\sqrt{30}}=\;-1.57$$

**Figure 5:**
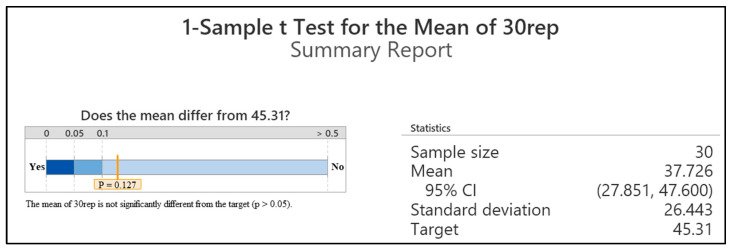
Comparison between the real system and the simulation model.

For the two-sided *t*-test, if $\left|t_{0}\right|>t_{\propto /2,n-1}$, the null hypothesis $H_{0}$ is rejected, and the model is considered inappropriate for predicting patient waiting times. However, since $\left|t\right|=1.57\;<t_{0.025,29}=2.045$, $H_{0}$ is accepted. Since the observed average waiting time of the real system (45.31 min) lies within the confidence interval obtained from the simulation outputs, the simulation model is considered to be statistically consistent with the observed system performance and results shown in Figure 5. The horizontal scale represents the *p*-value regions used for hypothesis testing. The dark blue zone (*p* < 0.05) indicates the rejection region of the null hypothesis, while the lighter zones (*p* > 0.05) represent non-significant regions. The calculated *p*-value (0.127) falls within the non-significant region, indicating that the mean of the 30 replications is not statistically different from the target value of 45.31.

It should be noted that the relatively high standard deviation reflects the inherent stochastic variability of patient arrivals and service processes in the hospital environment. In healthcare systems, waiting time distributions often exhibit considerable variability due to fluctuating demand and queue formation.

Following the validation stage, the required number of replications was determined in order to improve the precision of the estimated performance measures. Using the half-width estimation approach and the variance obtained from the preliminary simulation runs, the number of replications required to reduce the variability of the estimated average waiting time was calculated. Initially, 30 replications (R_0_) will be used to find $S_{0}^{2}$, which is the variance of the initial population. Half-width (h.l.) will be calculated with the following formula:

$$h.l.=\frac{t_{\propto /2,R-1}S_{0}}{\sqrt{R}}\;\leq \;\in$$

To find the R value,

$$R\geq {\left(\frac{t_{\propto /2,R-1}S_{0}}{\in }\right)}^{2}$$

If aiming to reduce the variability in a patient’s AWT by 70% with a probability of 0.95, the expected half-width should be 5.11 min, and the number of replications within which this variability can be achieved is

$${\left(\frac{t_{0.025,9}S_{0}}{\in }\right)}^{2}={\left(\frac{\left(2.045\right)\left(26.44\right)}{5.11}\right)}^{2}=111.96$$

The analysis indicated that when the number of replications is increased to 112, the standard error of the average waiting time decreases to 2.79 min, resulting in a 95% confidence interval of (35.35, 46.39) minutes. This represents a substantial improvement in the precision of the estimated performance measures.

**Figure 6:**
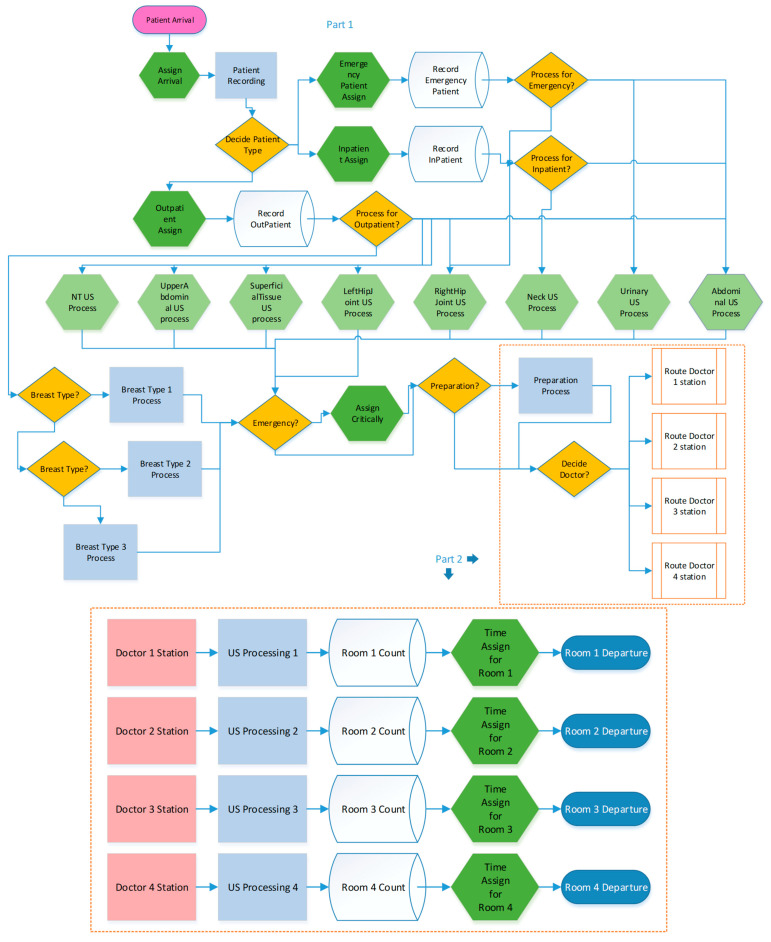
Simulation model for the US department by considering the current situation.

Figure 6 presents the simulation model, which represents the current state of the hospital. This component of the model determines the type of ultrasonography required for each incoming patient and assigns the corresponding processing time. The current working design of the department is implemented as the second part of the simulation model and is shown in Figure 6.

### 3.4. Comparative Analysis of Different Scenarios

After testing the model’s accuracy, we created alternative scenarios for the usage of IoT applications and different US department designs. In healthcare, the IoT emphasizes the role of integrating physical objects with computer systems to collect and process data, leading to improved services and efficiency. IoT technology enhances patient care, reduces costs, and improves overall healthcare efficiency. Wearable sensor technology has been explored as an IoT application that allows patients to conduct a breast ultrasound in the comfort of their own homes before visiting the hospital.

The simulation outputs provide valuable insight into the operation of the system, allowing us to determine its initial performance values. Patient wait time, an important performance criterion, was determined to be 35.95 min under the current conditions (S1). The aim of this study is to enhance the operational system of the ultrasound department to improve performance values under different scenarios. Six different operational scenarios were evaluated in the simulation study. Scenario 1 represents the current system configuration with four physicians. Scenario 2 corresponds to the alternative system design under the same staffing level of four physicians, and Scenario 3 corresponds to the configuration where breast examinations are not performed in the US department, with four physicians using IoT devices within the existing system structure. Scenario 4 represents an alternative system with four physicians and breast examinations performed using IoT devices. Furthermore, to examine the impact of reduced staff numbers, Scenario 5 reflects the existing system with three physicians, while Scenario 6 represents an alternative system configuration with three physicians. The goal is to minimize the waiting time for patients by making changes to the operational system of the ultrasound department and utilizing IoT applications. Another significant criterion is minimizing the idle time of staff. To address these two important criteria, a different structure was considered for the current operational system of the ultrasound department, leading to the creation of the second scenario (S2). In S2, a pooled resource policy is implemented, in which physicians are dynamically assigned to whichever examination room becomes available, and no fixed doctor–room pairing is enforced. This approach allows for resource optimization. The system’s performance was tested using IoT applications in both the current situation (S3) and the proposed second scenario (S4). It was assumed that the use of an IoT-based system for breast examinations would lead to a 30% reduction in the number of patients presenting for hospital-based examinations. Among the patients who are still present for examination, 70% are assumed not to use the IoT system and therefore undergo the examination with standard processing times. The remaining 30% are assumed to visit the hospital after detecting a suspicious condition through the IoT system. Among these patients, 50% are expected to have a shorter examination time because the location of the lesion has already been identified, while the other 50% are assumed to require a longer examination time due to the need for more detailed evaluation. Based on these assumptions, the scenarios were developed and the simulation model was adjusted accordingly. Additionally, the third scenario considers the use of IoT applications in the current situation, while the fourth scenario evaluates the use of the IoT in the proposed second scenario. All scenarios were run with 112 replications, and the results obtained were statistically analyzed.

After assessing the model’s accuracy, an alternative scenario was developed. This scenario considers the potential of conducting breast ultrasound examinations in patients’ homes through wearable sensor technology enabled by recent technological advancements. It is incorporated to evaluate how the integration of such technologies—if approved and implemented within the healthcare system—could affect operational performance metrics. The aim is to assess potential system-level impacts under a reconfigured service structure supported by these emerging technologies. The defined scenarios are as follows:

Scenario 1 (S1): Current system with the baseline doctor schedule (4 doctors until 2:00 P.M., 3 doctors afterward).
Scenario 2 (S2): Alternative system with the baseline doctor schedule.
Scenario 3 (S3): IoT-supported system with the baseline doctor schedule.
Scenario 4 (S4): Alternative system with IoT support and the baseline doctor schedule.
Scenario 5 (S5): Current system with three doctors available throughout the working day.
Scenario 6 (S6): Alternative system with three doctors available throughout the working day.

**Figure 7:**
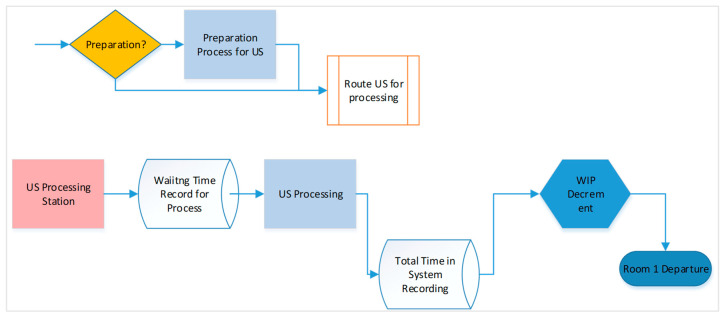
The second part of the alternative system of the simulation model.

Based on the current system model, Scenario 2 introduces an alternative system by modifying the second part of the simulation model, as shown in Figure 7. All alternative systems defined in the above scenarios are evaluated using this simulation model.

#### 3.4.1. Comparison of All Performance Metrics

**Figure 8:**
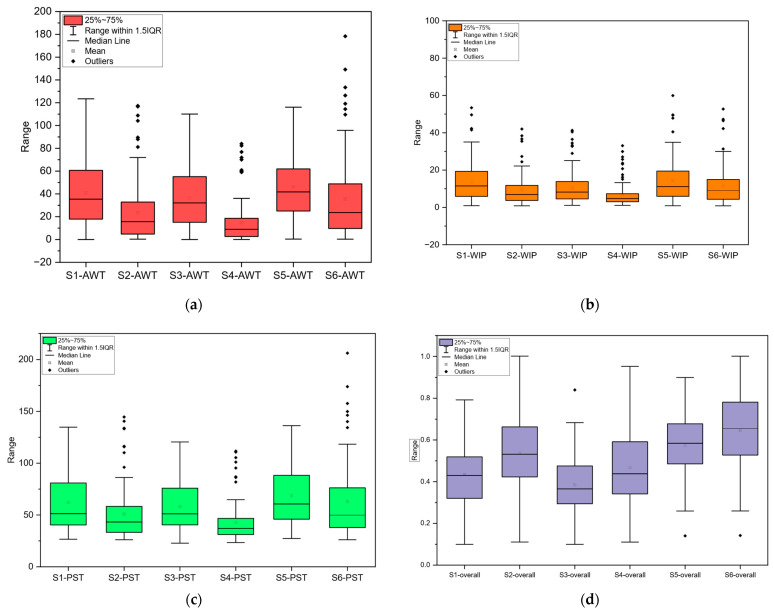
Comparison of all scenarios. (**a**) Average waiting time (AWT); (**b**) Work-in-process (WIP) for scenarios; (**c**) Average amount of time patients spends (PST) in US department; (**d**) Utilization rate of doctors for scenarios.

Figure 8 shows the average patient waiting time for all scenarios and the variability of waiting time at a 95% confidence level. To statistically evaluate whether the observed differences among scenarios were significant, a one-way ANOVA was conducted for each performance indicator, including waiting time, work-in-process (WIP), patient time in system (PST), and resource utilization. The ANOVA results indicated statistically significant differences among scenario means (*p* < 0.05). Therefore, post hoc multiple comparison tests were performed using both Tukey’s HSD and Fisher’s LSD procedures to identify pairwise differences between scenarios. All statistical analyses were conducted in full using OriginPro v2026 and Minitab 2026. The post hoc analysis showed that Scenario 4 achieved significantly lower mean values for waiting time, WIP, and PST compared to most alternative scenarios. However, when excluding configurations that incorporate technology-supported processes, Scenario 2 emerged as the most favorable operational alternative in terms of these performance indicators. Regarding resource utilization, Scenario 6 produced the highest average utilization rate, whereas Scenario 3 resulted in the lowest utilization among all scenarios. These findings indicate that performance improvements differ depending on the selected evaluation criterion, highlighting the trade-offs between efficiency and capacity utilization.

As a result of the evaluation of these data, the alternative scenario can provide an average reduction of 42% compared to the current scenario. If the current structure is maintained and breast ultrasound, which is an IoT application, is not performed in the hospital, this rate will be 12.18%. If both the alternative system is switched and the breast IoT application is used, there may be a significant reduction in waiting time (63.78%).

In Table 6 and Figure 8, the changes in all scenarios can be seen. Figure 8a illustrates the mean waiting time values obtained in all scenarios. Accordingly, Scenarios 2, 4, and 5 are completely different from the other scenarios. Scenario 5 has the longest waiting time, and scenario 4 has the shortest. In Scenario 5, the current situation in the hospital without a 4th doctor working part-time is considered. Scenario 2, which was obtained with a different alternative design, has the second shortest waiting time. In Scenario 4, both the alternative system and the situation where breast ultrasound is performed with an IoT solution are considered. Figure 8b shows the WIP and grouping results of each scenario. Scenario 4 contains the minimum number of patients waiting between processes. In the current system, assuming that three doctors are working, the number of patients waiting is 14.28, whereas when the alternative system with 3 doctors is considered, the number of patients waiting is forecasted decrease to 9.45. The simulation results regarding the average PST are shown in Figure 8c.

In addition to minimizing waiting times, it is also important to decide the optimum number of resources. Mistakes made in resource planning can cause waiting times to increase. In this study, since we are currently working with 4 doctors, 4 doctors were considered in the first 4 scenarios in simulation modeling. In the 5th and 6th scenarios, the situation of working with 3 doctors was analyzed. For the current scenario (S1), it can be observed that UtilADC has an average of 43%. Doctors’ utilization rates in alternative scenarios were examined, and the results are summarized in Figure 8d. If Scenario 6 (S6)—an alternative to the current situation featuring 3 doctors—were adopted, the average doctor utilization rate would increase to 57%. With simulation modeling, performance criteria that will help the hospital management make decisions about the operating system of the US department were obtained. The system that best minimizes patient waiting time can be achieved by implementing the proposed alternative system with 4 doctors and using IoT solutions. Alternatively, in the proposed system, it is considered that doctors are allocated to rooms according to the patient’s arrival and waiting status, instead of sitting in fixed, pre-assigned rooms.

#### 3.4.2. Decision of the System for the US Department

In decision-making tasks like the one presented in this paper, problems can arise due to the fact that multiple alternative scenarios are being evaluated, the simultaneous improvement of multiple performance indicators, and the variation in the analytical approaches employed. These problems are also inherently complex due to the conflicting nature of performance indicators, which prevents the existence of a single globally optimal solution. In other words, an improvement in one performance measure may lead to regression in another, thereby increasing the complexity of the decision-making process.

In this study, multiple system performance indicators were analyzed, including the average patient waiting time, the total time spent in the system, the number of patients waiting in the system, and physicians’ utilization rates. The number of physicians constitutes a key decision variable that directly affects the remaining performance indicators. While an increase in the number of physicians leads to a reduction in patient waiting times, real-world healthcare systems operate under limited resource constraints, and any additional resource allocation results in increased operational costs. To address this trade-off and to support the selection of the most appropriate scenario, the TOPSIS method by Hwang and Yoon [29], one of the most widely used multi-criteria decision-making techniques, was applied. When making decisions using the TOPSIS method, the selected alternative is expected to be close to the ideal solution and far from the negative-ideal solution. If the objective is to maximize return, closeness to the ideal solution implies maximization of benefit, whereas distance from the negative ideal solution corresponds to minimization of cost. In TOPSIS, the preferred alternative is the one that is nearest to the ideal solution and farthest from the negative ideal solution. The steps of the method are summarized in Refs. [30,31,32]:

Step 1. The decision matrix (A) consists of decision alternatives (m) in the rows and evaluation criteria (n) in the columns. The matrix has dimensions m×n and is denoted by $a_{ij}$.

Step 2. Each element $a_{ij}$ in the decision matrix is normalized. First, the sum of squares of the values in each column is calculated. Then, each $a_{ij}$ value is divided by the square root of the corresponding column sum of squares. The resulting normalized matrix is denoted by $n_{ij}$ (matrix N).

$$n_{ij}=\frac{a_{ij}}{\sqrt{\sum_{i=1}^{m}a_{ij}^{2}}},\;i=1,\ldots ,m;j=1,\ldots ,n$$

Step 3. Each element of the normalized matrix is multiplied by its corresponding criterion weight $w_{i}$. The weights are assigned according to the relative importance of the criteria and must be equal to 1. They typically take values between 0 and 1. The resulting weighted normalized values are denoted by $v_{ij}$; the matrix V is calculated as follows:

$$v_{ij}=w_{i}{\times n}_{ij}$$

Step 4. After obtaining the weighted normalized matrix (V matrix), the ideal and negative ideal solutions are identified based on the problem structure. If the objective is maximization, the maximum value in each column represents the ideal solution value, while the minimum value in each column represents the negative ideal solution value. If the objective is minimization, the reverse procedure is applied.

The notation is expressed as follows:

Ideal solution:

$$A^{+}=\{v_{1}^{+},\ldots ,v_{n}^{+}\}$$

$$v_{i}^{+}={max}_{i}\left\{v_{ij}\right\},i=1,\ldots ,m;\;j=1,\ldots ,n$$

Negative ideal solution:

$$A^{-}=\{v_{1}^{-},\ldots ,v_{n}^{-}\}$$

$$v_{i}^{-}={min}_{i}\left\{v_{ij}\right\},i=1,\ldots ,m;\;j=1,\ldots ,n$$

Step 5. The separation distance measure is used to calculate the distance of each alternative from the ideal and negative ideal points. The aim is to identify the alternative with the smallest distance to the ideal solution and the largest distance from the negative ideal solution.

The distances are computed as:

Ideal distance:

$$S_{i}^{+}=\;\sqrt{\sum_{j=1}^{n}{\left(v_{ij}-v_{i}^{+}\right)}^{2}},\;i=1,\ldots ,m;\;j=1,\ldots ,n$$

Negative ideal distance:

$$S_{i}^{-}=\;\sqrt{\sum_{j=1}^{n}{\left(v_{ij}-v_{i}^{-}\right)}^{2}},\;i=1,\ldots ,m;j=1,\ldots ,n$$

Step 6. The relative closeness of each alternative to the ideal solution is calculated using the distances obtained above. This measure is denoted by $C_{i}$.

$$C_{i}=\frac{S_{i}^{-}}{S_{i}^{-}+S_{i}^{+}},\;i=1,\ldots ,m,\;0<C_{i}<1$$

Step 7. Finally, alternatives are ranked in descending order based on $C_{i}$ values.

In this study, the considered alternatives represent different scenarios, while the decision criteria correspond to key performance indicators (KPIs). The criteria include average waiting time (AWT), work-in-process (WIP), patient spent time (PST), and utilization rate (UtilADC). AWT, WIP, and PST are minimized, whereas UtilADC is maximized. The problem includes four decision criteria and six alternatives. The simulation results are presented in Table 6. The average values reported in Table 6 constitute the decision matrix. After normalization, the normalized decision matrix is obtained. Subsequently, weights reflecting the relative importance of the criteria are assigned and multiplied with the normalized matrix. In this study, alternative rankings were analyzed under different weighting scenarios. The results obtained using different weight combinations are summarized in Table 7.

If equal weights are assigned to all criteria, Scenario 4 yields the best result. This scenario is applicable if the IoT application is implemented; otherwise, Scenario 2 should be selected as the next best alternative. When a higher weight is assigned to the AWT criterion, Scenario 4 again ranks first, followed by Scenario 2. However, if greater importance is given to the overall utilization rate, Scenario 2 becomes the best-performing alternative. These findings indicate that the final decision should be made in accordance with the relative importance assigned to the criteria within the hospital’s policy framework.

## 4. Conclusions

While previous studies have focused on patient appointment scheduling systems and determining appointment intervals, this study addresses patients entering the US department without an appointment (walk-ins). Detailed data analysis was performed based on a specific hospital’s historical data, and the necessary parameters for simulation modeling were determined. An analysis of the current situation was conducted, as was simulation modeling. Different scenarios were designed to improve performance values. The contributions of IoT applications recently used for disease diagnosis in the ultrasound department were examined.

This study also analyzed the impact of the IoT on hospital services, specifically its effect on patient waiting times, work-in-progress, patient time spent in the system, and utilization, which are key performance metrics. The evaluation was conducted using a simulation method. Additionally, a wearable breast ultrasound device, recently studied by Du et al. [21], was utilized to assess waiting times and other performance metrics for patients in the US department. This analysis showed that the AWT for patients in the US department decreased by 10.9%. Furthermore, there could be a 23% reduction in the WIP between 9:00 and 18:00 on weekdays. These improvements are expected with the implementation of IoT applications within the hospital system, which will provide hospital management with a clear understanding of the expected outcomes. This study demonstrates that implementing alternative working systems in the ultrasound department leads to improvements in performance indicators. Furthermore, various scenarios were simulated, and an approach was proposed to support decision-making based on comparative performance evaluation.

This study examined various performance indicators. Simulation optimization was found to be the best method to determine the appropriate number of resources to achieve the desired goal. Future studies will explore different scenarios to improve the system by considering physicians’ working hours and incorporating cost and profitability factors. Additionally, with the increasing use of smart watches and other wearable health technologies, certain diagnostic or monitoring procedures that do not require physical hospital visits may be increasingly performed. The limitations of this study and areas requiring further development include the fact that the use of IoT devices in healthcare is not yet widespread, and their accuracy levels following large-scale application have not been sufficiently validated. Therefore, it is recommended that accuracy rates and the potential need for repeated procedures be included in future studies. Thus, future research should focus on conducting feasibility analyses of such technology-assisted healthcare configurations and ensuring their reliability and practical applicability.

## Figures and Tables

**Table 1:** Yearly data regarding patients arriving per day in the US department.

Variable	Year	Mean	SE Mean	StDev	Minimum	Q1	Median	Q3	Maximum
Patient number	2021	44.44	3.79	18.59	20.30	25.34	40.88	63.12	66.09
	2022	41.41	3.39	16.62	19.30	25.19	40.37	58.00	62.00
	2023	39.28	4.32	16.16	20.30	22.81	38.83	54.67	60.70

**Table 2:** Descriptive statistics for patient arrivals according to day of the week (weekday or weekend day).

Variable	Weekday/Weekend Day	N	Mean	SE Mean	StDev	Minimum	Q1	Median	Q3	Maximum
Patient Number	Weekday	43	58.41	1.16	7.61	29.05	54.18	60.55	63.23	74.07
	Weekend day	43	25.09	0.829	5.44	8.125	22.50	25.25	29.000	35.89

**Table 3:** ANOVA for hours.

Sources	Degrees of Freedom	Adj. SS	Adj. MS	F-Value	*p*-Value
Hours	23	10480	455.631	655.57	0.000
Error	1907	1325	0.695		
Total	1930	11805			

**Table 4:** Interarrival time distribution for every time period.

Time Period	Interarrival Time Distribution (min.)	*p*-Value
8 A.M.–9 A.M.	52 × Beta (0.576, 2.12)	0.11
9 A.M.–10 A.M.	−0.5 + Gamma (9.33, 0.851)	0.298
10 A.M.–11 A.M.	54 × Beta (0.488, 2.34)	0.578
11 A.M.–12 P.M.	−0.5 + 54 × Beta (0.729, 3.86)	0.074
12 P.M.–1 P.M.	−0.5 + 28 × Beta (0.669, 1.48)	0.234
1 P.M.–2 P.M.	−0.5 + 56 × Beta (0.451, 1.63)	0.578
2 P.M.–3 P.M.	54 × Beta (0.488, 2.34)	0.727
3 P.M.–4 P.M.	Expo (11.7)	0.409
4 P.M.–5 P.M.	−0.5 + 61 × Beta (0.636, 1.95)	0.067
5 P.M.–6 P.M.	−0.5 + 61 × Beta (0.639, 1.21)	>0.75
6 P.M.–11 P.M.	Expo (56.9)	>0.75
11 P.M.–1 A.M.	Expo (39.6)	0.672
1 A.M.–7 A.M.	UNIF (1, 358)	>0.75

**Table 5:** Distribution of US processing types.

US Types	Processing Time Distribution (min.)	Number of Patients	Percentage
Abdominal Ultrasound	9 + Expo (5.69)	14,475	36.1
Urinary System Ultrasound	9.5 + 11 × Beta (0.802, 1.36)	5221	13.0
Bilateral Breast Ultrasound	UNIF (15, 30)	4504	11.2
Neck Ultrasound	9.5 + 11 × Beta (0.857, 1.66)	3154	7.9
Right hip joint Ultrasound	4.5 + Expo (2.92)	3141	7.8
Left hip joint Ultrasound	4.5 + Expo (2.92)	2517	6.3
Superficial Tissue Ultrasound	5 + Gamma (1.59, 3.26)	2467	6.1
Thyroid Ultrasound	UNIF (5, 18)	2221	5.5
Upper Abdominal Ultrasound	5 + Gamma (1.59, 3.26)	1423	3.5
Nuchal Translucency Measurement	10 + Erlang (3.75, 2)	1013	2.5

**Table 6:** Summary of simulation results for all scenarios.

Performance Metrics	Scenario 1 (Current)		Scenario 2		Scenario 3		Scenario 4		Scenario 5		Scenario 6	
	Avg.	Half-Width of 95% C.I.	Avg.	Half-Width of 95% C.I.	Avg.	Half-Width of 95% C.I.	Avg.	Half-Width of 95% C.I.	Avg.	Half-Width of 95% C.I.	Avg.	Half-Width of 95% C.I.
AWT (min)	40.87	5.52	23.68	4.82	36.43	4.88	14.80	3.46	46.28	5.22	35.56	6.52
WIP	13.55	1.87	9.45	1.49	10.43	1.59	6.63	1.14	14.28	2.10	11.49	1.87
PST (min)	62.17	5.16	50.98	4.76	58.02	4.23	42.87	3.43	68.68	5.41	63.01	6.46
UtilDC1	0.64	0.03	0.58	0.03	0.58	0.03	0.50	0.03	0.64	0.03	0.65	0.04
UtilDC2	0.45	0.02	0.56	0.03	0.39	0.02	0.49	0.03	0.45	0.02	0.64	0.04
UtilDC3	0.4	0.03	0.57	0.03	0.31	0.03	0.48	0.03	0.62	0.04	0.64	0.04
UtilDC4	0.25	0.06	0.45	0.04	0.26	0.05	0.41	0.04	-	-	-	-
UtilADC	0.43	0.03	0.54	0.03	0.39	0.03	0.47	0.03	0.44	0.03	0.57	0.04

Avg.: average; AWT: average waiting time; WIP: work-in-process; PST: time patients spend in the US department; UtilDC1: Utilization of Doctor 1; UtilDC2: Utilization of Doctor 2; UtilDC3: Utilization of Doctor 3; UtilDC4: Utilization of Doctor 4; UtilADC: Utilization of all doctors.

**Table 7:** Scenarios ranking based on the TOPSIS approach.

Alternative Scenarios	Weights for KPIs	TOPSIS			
	(AWT, WIP, PST, ADC)	$S_{i}^{+}$	$S_{i}^{-}$	$C_{i}$	Rank
S1	(0.25, 0.25, 0.25, 0.25)	0.109	0.023	0.173	5
S2		0.040	0.091	0.696	2
S3		0.086	0.049	0.362	4
S4		0.022	0.126	0.853	1
S5		0.127	0.011	0.082	6
S6		0.083	0.057	0.406	3
S1	(0.60, 0.10, 0.10, 0.20)	0.188	0.039	0.173	5
S2		0.064	0.163	0.718	2
S3		0.157	0.072	0.313	3
S4		0.017	0.226	0.929	1
S5		0.226	0.009	0.039	6
S6		0.149	0.083	0.357	4
S1	(0.20, 0.10, 0.10, 0.60)	0.097	0.027	0.215	4
S2		0.030	0.095	0.763	1
S3		0.107	0.028	0.207	6
S4		0.052	0.091	0.637	3
S5		0.104	0.027	0.208	5
S6		0.054	0.097	0.642	2

## Data Availability

The dataset cannot be made fully publicly available due to confidentiality and data protection considerations. The subset of the dataset may be made available upon reasonable request to the corresponding author, subject to appropriate conditions.
